# Faster SCDNet: Real-Time Semantic Segmentation Network with Split Connection and Flexible Dilated Convolution †

**DOI:** 10.3390/s23063112

**Published:** 2023-03-14

**Authors:** Shu Tian, Guangyu Yao, Songlu Chen

**Affiliations:** School of Computer & Communication Engineering, University of Science and Technology Beijing, Beijing 100083, China

**Keywords:** semantic segmentation, real-time, split connection, flexible dilated convolution

## Abstract

Recently, semantic segmentation has been widely applied in various realistic scenarios. Many semantic segmentation backbone networks use various forms of dense connection to improve the efficiency of gradient propagation in the network. They achieve excellent segmentation accuracy but lack inference speed. Therefore, we propose a backbone network SCDNet with a dual path structure and higher speed and accuracy. Firstly, we propose a split connection structure, which is a streamlined lightweight backbone with a parallel structure to increase inference speed. Secondly, we introduce a flexible dilated convolution using different dilation rates so that the network can have richer receptive fields to perceive objects. Then, we propose a three-level hierarchical module to effectively balance the feature maps with multiple resolutions. Finally, a refined flexible and lightweight decoder is utilized. Our work achieves a trade-off of accuracy and speed on the Cityscapes and Camvid datasets. Specifically, we obtain a 36% improvement in FPS and a 0.7% improvement in mIoU on the Cityscapes test set.

## 1. Introduction

**Figure 1:**
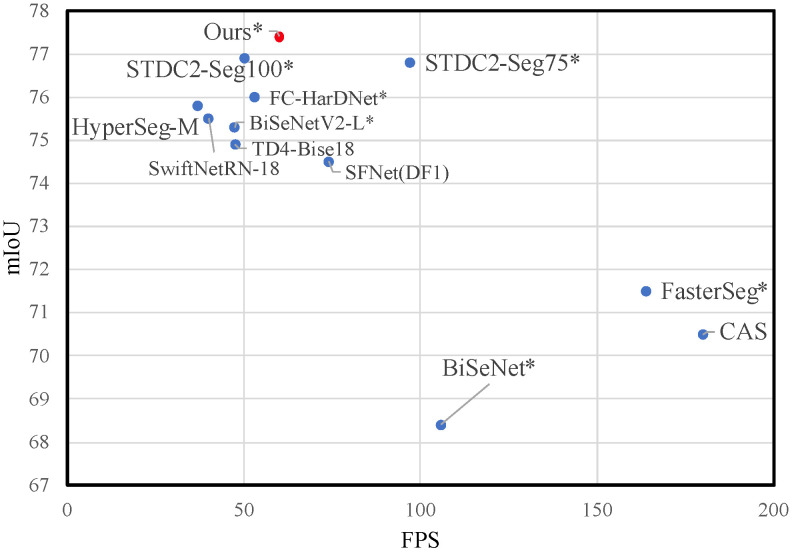
Speed-accuracy performance comparison on the Cityscapes test set. The red dot indicates our work while the blue dot indicates other methods. Our approach achieves the best speed-accuracy trade-off. The corresponding speed is measured using tensorrt acceleration if the method is marked with asterisk.

Semantic segmentation is a fundamental task in computer vision, which aims to perform pixel-level classification of a given image. The rapid development of deep learning has improved the accuracy and speed of semantic segmentation. Many deep learning networks have been deployed on edge devices, but the network of high-resolution segmentation is difficult to design. The inferenc speed need to increase further. As shown in Figure 1, the popular methods trys to get a trade-off between accuracy and speed.

A significant amount of work [1,2,3] has focused on designing decoders of semantic segmentation while fully transplanting state-of-the-art backbones from the object detection task. DeepLabV3 [4] uses ResNet-101 [5] as backbone to extract features. Many of these approaches achieved state-of-the-art segmentation accuracy because they used a deep and wide CNN backbone and did not limit computation costs, which creates a bottleneck in inference speed.

Meanwhile, semantic segmentation has its own requirements for feature extraction, as the specialized backbone networks have the advantage of segmentation accuracy. To further improve segmentation accuracy, much real-time semantic segmentation [6,7,8,9] work has focused on redesigning backbone networks for the task. These backbones all pay attention to the balance between spatial information and large receptive fields. Most of them contain various forms of feature aggregation modules or structures to make up for the loss of low-level information. FCN [10] proposed a skip connection and then PSPNet [11] proposed a pyramid pooling module, which preserves too many redundant features. They simply concatenate features with different resolutions, resulting in the redundancy of feature widths in the network. Then, some researchers propose different dense connections [1,12,13,14] to improve the efficiency of gradient propagation in the network so that more useful and advanced features can be perceived and deeper networks can be trained. Most of these approaches use a single-path dense connection structure and achieve a satisfactory trade-off. However, in the inference phase, this lightweight single-path down-sampling network does not have any space for parallel optimization. These single-path dense connection networks’ speeds are still limited. The decoder is another key element for real-time segmentation. Multi-scale information is very helpful but very time-consuming.

Therefore, we propose a dual-path backbone SCDNet and a lightweight decoder to improve the inference speed and segmentation accuracy. Specifically, we propose a split connection (SC) and three-level hierarchical module (THM) to build a dual-path structure, which introduces parallelism in the inference phase and preserves more low-level features for the decoder. Secondly, we propose a flexible dilated convolution (FDC) in the high level of the convolutional layers. It uses variable dilation rates to enrich the receptive field size. The FDC can self-select receptive fields of different sizes to improve the segmentation accuracy of objects of certain sizes. Finally, we refine the Flexible and Lightweight Decoder (FLD) [15] to reduce computation cost. Our method increases inference speed by proposing a dual path lightweight backbone that can be plugged into different frameworks while improving segmentation accuracy significantly.

Our work contributions can be summarized as follows:

- We propose a split connection structure to complement more low-level features for the output while introducing parallelism to improve the inference speed. We also propose a three-level hierarchical module to fuse the features of three resolutions;
- We propose a flexible dilated convolution to adjust the receptive field of the network and enrich the size of the receptive field of the output;
- We refine the flexible and lightweight decoder to improve computation speed and segmentation accuracy by utilizing multi-scale information fusing with a lightweight structure;
- We verify the effectiveness of the method on the Cityscapes and Camvid datasets. Specifically, we achieve a 36% improvement in FPS and a 0.7% improvement in mIoU on the Cityscapes test set.

(A preliminary version of the segmentation algorithm in this work appeared in the International Conference on Ubiquitous Intelligence and Computing 2022 by Yao et al. In this paper, we utilize a refined flexible and lightweight decoder to improve the performance of the method. Furthermore, we update the information of the experiments).

## 2. Related Work

Since semantic segmentation training is highly dependent on a high-precision annotated dataset, the unsupervised domain adaptation (UDA) semantic segmentation task has attracted significant attention. The recent UDA work DecoupleNet [16] introduces an auxiliary classifier to learn more discriminative target domain features. The over-fitting of the source domain is alleviated so that the segmentation model can be more focused on the segmentation task. However, in this paper, we do not focus on the unsupervised domain adaptation (UDA) semantic segmentation task.

There have generally been two types of semantic segmentation methods in recent years. One is semantic segmentation, which does not care about the computational cost. It often uses deeper and wider backbone networks and multi-scale methods, often using attention mechanisms or other ways to connect the output of multiple backbone networks. The other is real-time semantic segmentation, which makes a trade-off between accuracy and real-time speed in the mobile device. Lightweight backbone networks designed for semantic segmentation are often used, and the inference speed can reach several times that of ordinary semantic segmentation without much impact on the accuracy. In our method, the decoder is an important module; therefore, we introduce some related decoders besides the semantic segmentation method.

### 2.1. Semantic Segmentation

Since FCN [10] used CNN and a skip connection to solve the semantic segmentation task, various deep neural networks have emerged one after another. SegNet [17] adopted the FCN encoding–decoding architecture and used the unpooling operation in the upsampling process. UNet [18] introduced a U-shaped down-sampling and up-sampling structure. DeepLabV2 [4] proposed the Atrous Spatial Pyramid Pooling (ASPP) module to replace the pooling and convolution in PPM. HRNet [3] maintained a large resolution with three-scale branches and achieved state-of-the-art results in semantic segmentation despite being designed for human pose estimation. HMAS [2], which arranged three HRNet using the attention mechanism, pushed the accuracy of semantic segmentation to a new level.

### 2.2. Real-Time Semantic Segmentation

ENet [9] and some other works were the earliest to address real-time semantic segmentation. They began by reducing the number of down-samples, changing the convolution method, and introducing dilated convolution and asymmetric convolution to real-time semantic segmentation. The decoder–encoder architecture of ENet is unsymmetrical. The encoder is evidently larger than the decoder. ENet [9] achieved excellent results at that time. In recent years, the promising semantic segmentation neural architecture search (NAS) method breaks through the limitations of manual design. Auto-DeepLab [19] completely designed the search space of the network structure for the semantic segmentation task. Fasterseg [8] went further in the use of operators. Under the condition of a certain search space or hardware constraints, the design of network connection paths by NAS is far better than manual work. However, in network module design and complex topology design, manual design is temporarily difficult to be replaced. There are many manual networks that perform better than NAS.

RegSeg [20] proposes D block, and uses grouped convolution and dilated convolution at the same time to achieve state-of-the-art performance with a one-way encoder and simplified decoder. DDRNet [21] proposed the idea of bilateral fusion, using two resolution branches and only 23 layers of convolution layers to achieve high accuracy. Furthermore, the decoder of DDRNet is composed of numerous basic residual modules. At the same time, it is easy for these two works to infer high-definition video in real-time on 1080Ti. These two networks, respectively, illustrate the necessity of feature aggregation of high and low levels and enriching the receptive field.

The encoder–decoder architecture is a popular segmentation method. For the purpose to run in real time, the decoder is often a computationally efficient module, such as ENet [9] or DDRNet [21], as mentioned above. In this paper, we also adopt this architecture. We propose a split connection structure and a three-level hierarchical module to introduce a parallel structure and complement more low-level features for the output. In addition, we also introduce flexible dilated convolution into high-level convolutional layers to enrich the receptive field of the network. At last, a refined flexible and lightweight decoder is used.

## 3. Methods

### 3.1. Network Overview

**Figure 2:**
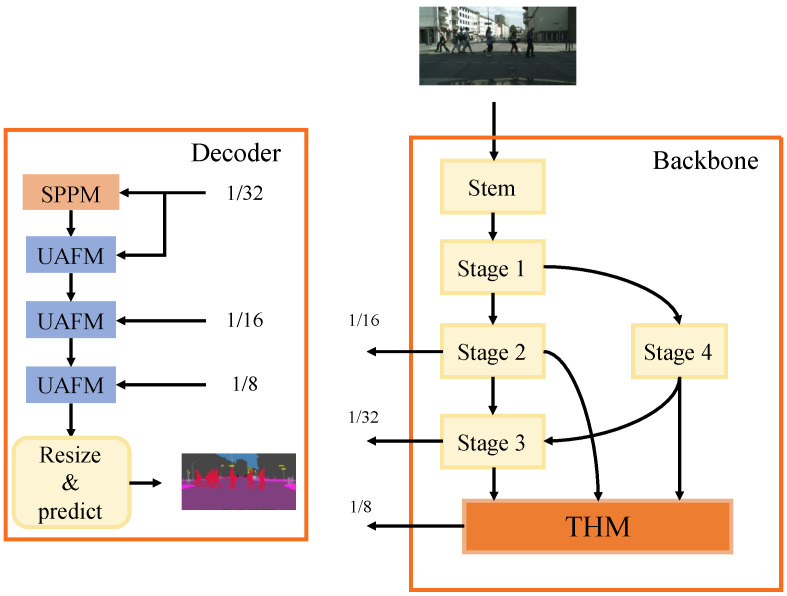
Overview of the network.

**Figure 3:**
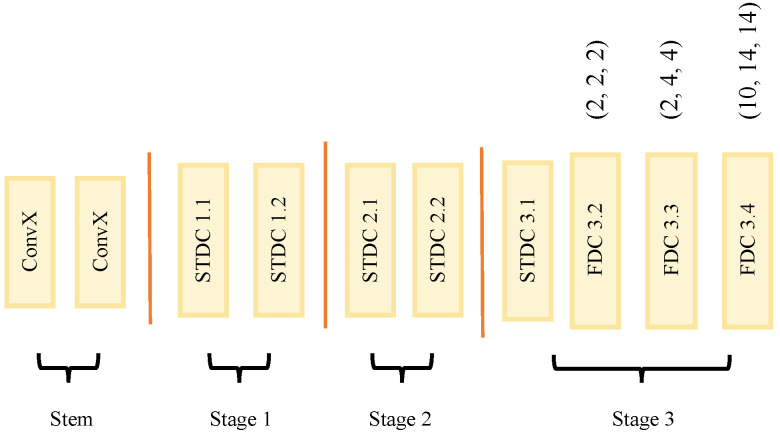
Main branch of backbone. It demonstrates the cascaded STDC blocks and FDC blocks. The three digits besides the FDC indicate the dilation rates of three ConvXD. The detailed information of FDC is shown in Figure 4.

The overview of the network is shown in Figure 2. The backbone consists of two branches, the main branch, and the auxiliary branch. The main branch structure is shown in Figure 3. The decoder is a refined, flexible, and lightweight decoder. The detailed guidance from STDC-Seg [12] is applied to improve the edge features extraction. First, we introduce the detail of the split connection structure and three-level hierarchical module (THM). Then, we present the whole architecture of our SCDNet backbone and the flexible dilated convolution. At last, a refined, flexible, and lightweight decoder is introduced.

### 3.2. Split Connection

The inside connection structure of the STDC backbone is shown in Figure 5a. In the main branch, to down-sample rapidly and ensure that the extracted semantic information is deep enough, it uses single-path deep convolutional layers. This concatenated structure leaves no room for optimization in inference acceleration. Meanwhile, in order to increase the fusion of high-level and low-level feature maps, the convolutional layers inside the STDC block are densely connected. Most of the single-path backbone outputs are features from high levels with large receptive fields and a lack of detailed information. Their perception of detailed information is poor.

Based on these two thoughts, we found that stages of different resolutions are only connected adjacently. Then, we attempted to connect Stage 1 and Stage 3 directly by dividing the feature maps channel-wise as shown in Figure 5b, and while the segmentation accuracy becomes worse under different ratios of channel concatenation, we believe that dividing channels results in losing important feature information, and them being unable to complement each other after concatenation. Directly adding features together is also unsatisfactory.

Therefore, we propose the split connection to connect Stage 1 and Stage 3 together to further enrich the connections. In order to ensure the low-level convolution depth and increase parallelism, the split connection method divides Stage 1 into two parts as shown in Figure 5c. The implementation process of our method is clearly shown in Figure 6. We first reduced the depth of Stage 2; then, we divided Stage 1 into two identical parts, and used the second part to connect Stage 1 and Stage 3. The following is an introduction to split connection implementation details.

The main branch consists of one stem and three-stage parts. The stem consists of two ConvX. ConvX is a basic convolutional unit, consisting of a convolutional layer, a batch norm layer, and a relu activation layer. The convolution kernels of the two ConvX here are both 3 × 3 and the stride is 2. The input image is first down-sampled by two layers of ConvX, and then fed into cascaded STDC blocks. This short densely connected blocks enhance the feature richness and maintain the network width to reduce computation costs. In the main branch, we use eight blocks. They are divided into three stages, which have two, two, and four blocks, respectively. Only the first block of each stage contains 1/2 down-sampling.

The output features of Stage 1 are fed into the auxiliary branch of the backbone. The auxiliary branch is composed of two blocks. The resolution of input and output features is 1/8, and the number of channels is 256. The output of the auxiliary branch is down-sampled by convolution with stride = 2 and added to the output of Stage 2. They are used as the input of Stage 3 together.

### 3.3. Three-Level Hierarchical Module

The three-level hierarchical module is inspired by DeepLabv3+ [22]. It can be understood as a small decoder to restore some detailed information. It is designed to hierarchically fuse features of three resolutions with little computation cost. The structure is shown in Figure 7. The features of 1/32, 1/16, and 1/8 go through a ConvX at the same time, and the output of 1/32 is up-sampled by bilinear interpolation and added to the output of 1/16. Then, it goes through a 3 × 3 ConvX and is up-sampled again. Finally, it concatenates with the output of the 1/8 feature. The feature resolution is fused in order from small to large. The following equations show the final difference between these two backbones.

(1) $$Output=S_{3}\left[S_{2}\left(S_{1}+S_{4}\right)\right]$$

(2) $$Output=S_{3}\left[S_{2}\left(S_{1}\right)+S_{4}\left(S_{1}\right)\right]+S_{2}\left(S_{1}\right)+S_{4}\left(S_{1}\right)$$

$S_{i}$ denotes *i*-th stage output or i-th stage itself. The outputs of Equations (1) and (2) show different connections of two backbones. The output of Equation (2) has richer high-level and low-level information of three resolutions than Equation (1).

The single-path method lacks low-level features at the output and structure for parallel inferring. We propose this dual-path backbone to improve the inference speed and preserve the high-resolution features.

### 3.4. Flexible Dilated Convolution

The receptive field (RF) refers to the range of the original image which a point in a certain layer of the network can perceive. From the perspective of the receptive field, a good segmentation network should preserve features with rich RF. For example, the perception of the vehicle requires the RF with vehicle size. The backbone should self-select receptive fields responding to its needs. Global average pooling is commonly used to obtain features of the entire image. Although global features can perceive all objects, the perception of objects is not accurate enough.

Therefore, we need to pursue a richer continuous receptive field and output features with large and small receptive fields, so that the network can have scalable respective fields and extract multi-scale information.

From the distribution of segmented objects, there are a few large objects whose size occupies half or more of the screen size. Therefore, using global average pooling or using too large convolution kernels to rapidly expand the receptive field will lead to the loss of certain size features and overlapping of image information. Therefore, we use different dilation rates of dilated convolution in Stage 3’s last three blocks to increase the receptive field. After the experiment, the inference delay added by using dilation convolution is very limited. However, a significant improvement can be achieved.

The formula for calculating the receptive field is as follows:

(3) $$l_{k}=l_{k-1}+\left[\left(f_{k}-1\right)*\prod_{i=1}^{k-1}s_{i}\right]$$

$l_{k}$ denotes the receptive field of the k-th layer, $f_{k}$ denotes the convolution kernel size, and $s_{i}$ denotes the stride of the i-th layer. The output of each block contains features with different sizes of RF. The following calculations of the receptive field focus on the largest receptive field.

Calculated by Equation (3), the maximum receptive field of the baseline backbone is 1199, which is almost a quarter of the maximum value that we need in Cityscapes [23] (4096). We hope that the maximum receptive field of the network can reach almost the entire image.

**Figure 4:**
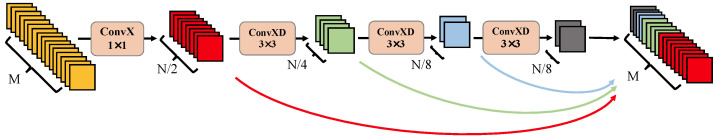
Structure diagram of flexible dilated convolution. ConvXD denotes ConvX with dilated convolution.

**Figure 5:**
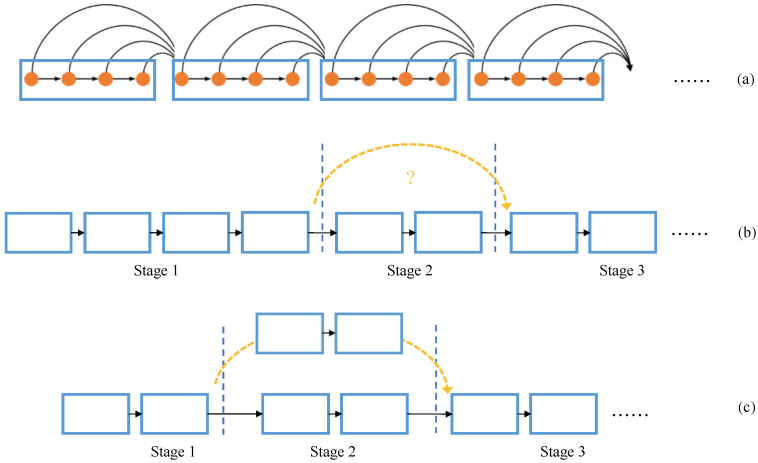
(**a**) STDC-Seg backbone, (**b**) split connection motivation, (**c**) our backbone. The orange circle denotes the convolutional layer, the blue rectangles denote the individual STDC blocks, and the yellow connection denotes the split connection.

**Figure 6:**
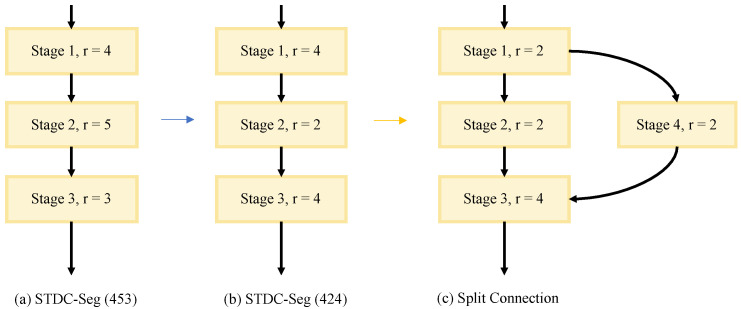
(**a**) Original STDC-Seg backbone, (**b**) backbone with simplified network depth, (**c**) split connection backbone. r denotes STDC block repeat time.

**Figure 7:**
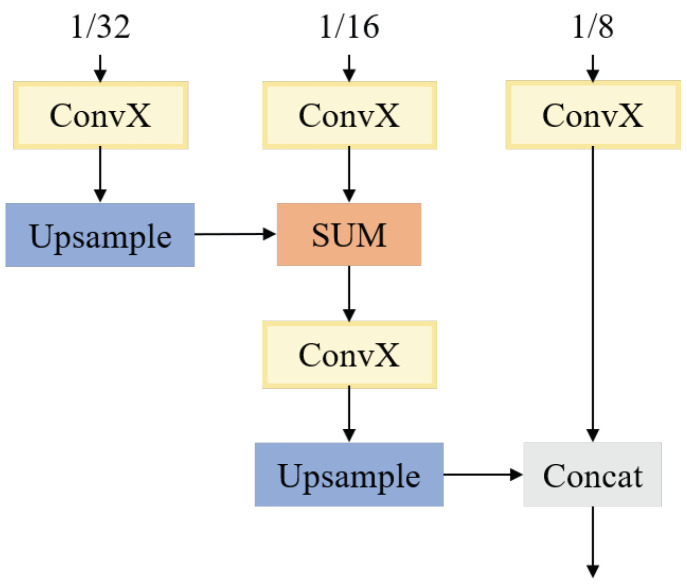
Three-level hierarchical module structure.

The baseline backbone can obtain four receptive fields of 1 × 1, 3 × 3, 5 × 5, and 7 × 7 in one basic block. Based on this combination, we introduce dilated convolution in the last stage of the backbone network, as shown in Figure 6b and Figure 4. The dilation rates are (2, 2, 2) (2, 4, 4) (10, 14, 14) in 1024 × 2048 images in Cityscapes. The inference delay will not be significantly increased, while the receptive field is increased to 3887 and covers almost the entire picture.

### 3.5. Refined Flexible and Lightweight Decoder

In order to improve the network’s perception of objects without significantly increasing computation costs, we use a refined flexible and lightweight decoder (RFLD), which combines the simple pyramid pooling module (SPPM) [15] and the unified attention fusion module (UAFM) [15]. As illustrated in Figure 2, we fuse three scales of features with the feature outputs by SPPM. Different from the original flexible and lightweight decoder [15], RFLD uses three UAFMs but not only the nethermost two UAFMs. As the final output of the encoder, the features with the down-sample ratio of 1/32 are very important. Therefore, it is not only utilized to generate features of SPPM, but also fused together.

#### 3.5.1. Simple Pyramid Pooling Module

In order to produce the refined feature, a real-time model, SPPM, is utilized. First, to obtain multi-scale information, three pyramid pooling modules whose bin sizes are 1 × 1, 2 × 2, and 4 × 4 are used. Then, all three features are aligned by convolution and resize. Finally, a refined feature is produced by adding all three up-sampled features and a convolution operation.

#### 3.5.2. Unified Attention Fusion Module

For fusing two level features, the unified attention fusion module (UAFM), which consists of the spatial attention module and the channel attention module, is trained to produce the weights. When the size of features are different, up-sampling is performed to align them.

The spatial attention module is simple and effective. As Equation (4) shows, mean operation and max operation are applied to the high-level feature and low-level feature. Then, these four features are concatenated. At last, convolution and sigmoid operations are utilized to produce a weight.

(4) $$\begin{array}{cc} F_{cat} & =Concat(Mean\left(F_{up}\right),Max\left(F_{up}\right),Mean\left(F_{low}\right),Max\left(F_{low}\right))\\ \alpha_{s} & =Sigmoid(Cov\left(F_{cat}\right) \end{array}$$

As Equation (5) shows, the channel attention module is similar to the spatial attention module. A total of four features are obtained by using average-pooling and max-pooling operations on the high-level feature and low-level feature. Then, it concatenates these four features and performs convolution and sigmoid operations to produce a weight.

(5) $$\begin{array}{cc} F_{cat} & =Concat(MeanPool\left(F_{up}\right),MaxPool\left(F_{up}\right),MeanPool\left(F_{low}\right),MaxPool\left(F_{low}\right))\\ \alpha_{c} & =Sigmoid(Cov\left(F_{cat}\right)) \end{array}$$

(6) $$F_{out}=\alpha *F_{up}+\left(1-\alpha \right)*F_{low}$$

Finally, as Equation (6) shows, the fused feature is the weighted sum of $F_{up}$ and $F_{low}$. Due to the fact that there is an evident spatial relation between the objects in the image, we exploit the inter-spatial relationship but not the inter-channel relationship. Therefore, we use the spatial attention module to generate the weight, that is to say, $\alpha =\alpha_{s}$.

## 4. Results

We use ISLVRC2012 [24] as a pre-training dataset for the downstream segmentation task and then train on Cityscapes [23] and Camvid [25] to verify and test the effectiveness and generalizability of our work, respectively. Next, we will introduce the dataset and metrics, train setting, ablation study, and comparison of different methods.

### 4.1. Datasets and Metrics

**ISLVRC2012** ISLVRC2012 [24] CLS has different sizes of images which are divided into 1000 classes. A total of 1,281,167 images are used for training, 50,000 images are used for validation, and 100,000 images without labels are used for testing.

**Cityscapes** Cityscapes [23] is one of the most commonly used semantic segmentation datasets. It contains 5000 pixel-level annotated images. Each image has a size of 3 × 2048 × 1024, with 2975 for the training set, 500 for the validation set, and 1525 for the test set. The annotation contains 30 categories, 19 of which are used for semantic segmentation training (ignore label = 255).

**Camvid** Cambridge-driving Labeled Video Database (CamVid) [25] provides 32 ground truth semantic labels. The size of the image is 3 × 960 × 720, and there are 701 finely annotated images that can be used for semantic segmentation, including 367 in the train set, 101 in the validation set, and 233 in the test set. We use both train and validation sets for training. There are 11 categories of commonly used semantic segmentation, and the rest are ignored labels (ignore label = 255).

**Metrics** In image classification, we use top-1 acc and top-5 acc as evaluation metrics. In the semantic segmentation task, we use class-wise intersection over union (mIoU) and frames per second (FPS) as the evaluation metrics.

### 4.2. Train Setting

In the image classification task, we follow the strategies proposed by HRNet [3]. We set max-epoch as 120, except that we change the segment head into a ConvX, a global average pooling, and a linear layer, and the whole network is used in the image classification task.

In the semantic segmentation task, we use the same hyper-parameters as STDC-Seg [12]. The batch size sets 12 for 4 GPUs in the Cityscapes dataset, and 24 for the Camvid dataset, respectively. We set the max iter 60,000, 10,000 for the Cityscapes dataset and CamVid dataset.

All experiments are conducted in ubuntu 18.04, torch 1.10.1 torch vision 0.11.2 on a docker. 4 RTX 2080Ti GPU and CUDA 10.1, CUDNN7.6.4 are used for training. One of them is used for inference experiments.

### 4.3. Ablation Study

This section presents ablation experiments to verify the effectiveness of each component in our method.

#### 4.3.1. Effectiveness of Flexible Dilated Convolution

**Figure 8:**
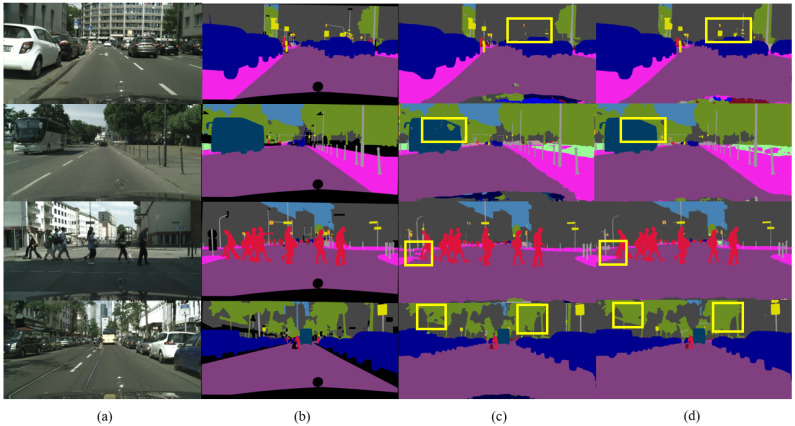
Comparisons between results of our work and results of baseline. The yellow rectangles mark the difference in segmentation accuracy between our method and baseline. (**a**) Denotes input images, (**b**) denotes ground truth, (**c**) denotes baseline, and (**d**) denotes our method.

As shown in Table 1, we significantly improved accuracy in most classes, while maintaining the same accuracy as the baseline in the other classes. As shown in Figure 8, we visualize the segmentation output. The road surface and vehicles in the images are segmented more completely.

Table 2 shows three groups of experiments we performed on dilatation rate combination, which were, respectively, used(1,1,1)(1,1,1)(1,1,1), (2,4,10)(2,4,10)(2,4,10), and (2,2,2)(2,4,4)(10,14,14) three dilatation rate combination. By calculating their RF, mIoU, FPS, and other data, the experiments show that the accuracy and speed of the last group of methods are the best.

#### 4.3.2. Effectiveness of Other Modules

To verify the effectiveness of split connection, the three-level hierarchical module (THM), and the refined flexible and lightweight decoder (RFLD), we train the networks with different components. Table 3 shows the accuracy and speed.

We can find that the split connection in the preliminary algorithm and final algorithm improves the mIoU by 0.5% and 0.6% respectively. Adding THM improves the mIoU by 0.2% in both versions of the algorithm.

RFLD is a lightweight decoder. However, it utilizes multi-scale information. When using RFLD as the decoder, we can find that there is a great enhancement of FPS; meanwhile, the accuracy is the same or slightly higher. The results show that split connection and THM could improve performance effectively and RFLD decreases the computational cost without performance reduction.

### 4.4. Results

#### 4.4.1. Comparison on Cityscapes

In Table 4, we present the accuracy and speed results of our network on Cityscapes. Due to the fact that the model uses different Gpus for inference, the FPS in the table are not completely comparable to each other. Our work makes a trade-off in accuracy and speed, which is 28.5% faster and 1.6% higher than the former SOTA FC-HarDNet-70 [1]. Our method is also 2.3% higher than BiSeNetV2 [7] in mIoU. We use full-size images and achieve 68.1 FPS and 77.6% in mIoU on the test set. Our baseline achieves 50.2 FPS and 76.9% in mIoU. We obtain a 36% improvement in FPS and a 0.7% improvement in mIoU.

Besides the typical methods, some recent works are shown in Table 4. CSRNet-heavy was published in 2023. It proposed the selective resolution module which assigns soft channel attentions across the feature maps and helps to remedy the problem caused by multi-scale objects The channel-wise attention method they use is inspiring, but the poor parallelism of the network leads to the limitation of reasoning speed. The test mIoU and FPS of CSRNet-heavy are lower than ours. The authors of MoSegNet-large do not publish test mIoU. As they also do not release the code, we only provide their published data. It is evident that our method is much faster than theirs.

#### 4.4.2. Comparison on Camvid

Table 5 below shows our work’s performance on the Camvid dataset. Our work is effective as well on Camvid. We achieve 154.2 FPS and 74.2% mIoU on the test set, which is better than STDC’s 123.5 FPS and 73.9%. Our method is also 3.1% ahead of the former SOTA [8].

## 5. Conclusions

Since the single-path dense connect backbone is not fast enough, we propose a split connection structure and THM to provide a parallel structure for parallel inference optimization and supplement low-level feature information. To eliminate the accuracy limitation of certain size objects, we introduce flexible dilated convolution to enrich the size of the network’s RF. Finally, a refined flexible and lightweight decoder is utilized to reduce the computational cost. Extensive experimental proofs and visualization results demonstrate the effectiveness of our work. In the future, we will continue to simplify the encoder and apply the networks to some specific scenes.

## Figures and Tables

**Table 1:** Three different receptive field networks’ per-class mIoU results on the Cityscapes val set.

Network RF	mIoU	Road	Swalk	Build.	Wall	Fence	Pole	Tlight	Sign	Veg	Terrain	Sky	Person	Rider	Car	Truck	Bus	Train	Motor	Bike
Baseline (1007/4096)	76.9	97.75	82.90	**92.32**	58.41	**62.32**	60.78	70.20	**77.29**	**91.90**	61.16	94.37	**79.99**	60.68	94.26	75.33	84.93	**80.17**	60.02	75.31
Ours (3503/4096)	77.3	97.83	82.90	92.28	56.96	61.53	62.26	**70.95**	77.26	91.67	59.64	94.38	79.75	**61.47**	94.66	79.42	87.20	79.83	**62.03**	76.17
Ours (3887/4096)	77.5	**97.87**	**83.06**	92.31	**58.54**	61.74	**62.32**	69.99	77.09	91.76	**61.81**	**94.57**	79.85	60.51	**94.72**	**81.24**	**87.43**	79.06	62.02	**76.26**

**Table 2:** Comparison of the effects of different maximum receptive fields size; RF denotes receptive field.

Backbone	Dilatation Rate Combination	RF	mIoU	FPS	GFLOPs
STDC1446	-	1199/4096	77.0	50.2	94.3
Ours	(1,1,1)(1,1,1)(1,1,1)	1007/4096	76.9	60.0	98.2
Ours	(2,4,10)(2,4,10)(2,4,10)	3503/4096	77.3	60.0	98.2
Ours	(2,2,2)(2,4,4)(10,14,14)	3887/4096	77.5	60.0	98.2

**Table 3:** Comparison of the accuracy and speed of the networks using different components on Cityscapes val set, the numbers behind the network denote the repetitions of STDC block, THM denotes three-level hierarchical module. RFLD denotes refined flexible and lightweight decoder. No RFLD means that the algorithm is in its preliminary version and the result is reported by our conference paper.

Network w/o TensorRT	FDC	Split Connection	THM	RFLD	mIoU	FPS	GFLOPs
STDC(424)					76.3	43.1	85.5
Ours(224)	*√*				76.8	44.7	76.6
Ours(224)	*√*	*√*			77.3	38.1	95.1
Ours(224)	*√*	*√*	*√*		77.5	35.4	98.2
Ours(224)	*√*			*√*	76.8	60.5	59.6
Ours(224)	*√*	*√*		*√*	77.4	50.1	78.1
Ours(224)	*√*	*√*	*√*	*√*	77.6	50.1	81.2
STDC1446(453)					77.0	37.4	94.3

**Table 4:** Comparison of the accuracy and speed of different networks on Cityscapes val set. * means using TensorRT.

Network	Val mIoU	Test mIoU	FPS	Resolution	Params(M)	GFLOPs	GPU
CAS [26]	71.6	70.5	108	768 × 1536	-	-	-
FasterSeg * [8]	73.1	71.5	163.9	1024 × 2048	4.4	28.2	GTX 1080Ti
MobileNetV3 [27]	72.4	72.6	-	1024 × 2048	1.51	9.74	GTX 1080Ti
BiSeNet * [6]	69.0	68.4	105.8	768 × 1536	5.8	14.8	GTX 1080Ti
BiSeNetV2-L * [7]	75.8	75.3	47.3	512 × 1024	47.3	118.5	GTX 1080Ti
SFNet(DF1) [28]	-	74.5	74	1024 × 2048	9.03	-	GTX 1080Ti
SFNet(DF2) [28]	-	77.8	53	1024 × 2048	10.53	-	GTX 1080Ti
CSRNet-heavy [29]	77.3	76.0	36.3	1024 × 2048	-	-	GTX 1080Ti
MoSegNet-large [30]	78.2	-	50.1	1024 × 2048	-	42	Titan RTX
FC-HarDNet-70 * [1]	77.7	76.0	53	1024 × 2048	4.12	35.6	Titan V
STDC2-Seg100 * [12]	77.0	76.9	50.2	1024 × 2048	16.1	94.3	RTX 2080Ti
Ours *	77.7	77.6	68.1	1024 × 2048	17.8	81.2	RTX 2080Ti

**Table 5:** Accuracy and speed results of different networks on Camvid dataset. * means using TensorRT.

Network	Resolution	Test mIoU	FPS
ENet [9]	720 × 960	51.3	61.2
BiSeNet * [6]	720 × 960	65.6	175
CAS [26]	720 × 960	71.2	169.0
FasterSeg * [8]	720 × 960	71.1	398.1
STDC2-Seg * [12] (Baseline)	720 × 960	73.9	123.5
Ours *	720 × 960	74.2	154.2

## Data Availability

Not applicable.
